# Adenine base editing of CFTR using receptor targeted nanoparticles restores function to G542X cystic fibrosis airway epithelial cells

**DOI:** 10.1007/s00018-025-05587-y

**Published:** 2025-04-07

**Authors:** Isabelle Rose, Miriam Greenwood, Matthew Biggart, Natalie Baumlin, Robert Tarran, Stephen L. Hart, Deborah L. Baines

**Affiliations:** 1 Institute of Infection and Immunity, School of Health and Medical Sciences, City St George’s, University of London, London, SW17 0RE UK; 2 Department of Genetics and Genomic Medicine, UCL Great Ormond Street Institute of Child Health, London, WC1N 1EH UK; 3 Division of Genetic, Environmental and Inhalational Disease, University of Kansas Medical Center, Kansas City, KS 66160 USA; 4 Department of Internal Medicine, University of Kansas Medical Center, Kansas City, KS 66160 USA

**Keywords:** Class 1 mutation, Transfection, Air–liquid-interface, Airway, Ciliated cell, Secretory cell

## Abstract

The cystic fibrosis (CF) causing variant G542X harbours a premature translation stop signal in the cystic fibrosis transmembrane conductance regulator (*CFTR*) mRNA. This results in nonsense-mediated decay and loss of functional CFTR protein which leads to defective anion transport and the development of CF disease pathology. Currently available CF modulator therapies cannot be used to treat this variant. We used an adenine base editor (ABE8e Cas9) and guide RNA (sgRNA)/enhanced green fluorescent protein (EGFP) plasmids encapsulated in receptor targeted nanoparticles (RTN), delivered to Bmi-1 transduced basal human CF airway epithelial cells harbouring the homozygous CFTR G542X variant, to convert the stop codon to G542R, a variant which is amenable to modulator therapy. ABE resulted in 17% of alleles edited to G542R and further selection of GFP fluorescent cells by FACS liberated a population with 52% G542R edited alleles with no editing of neighbouring adenines (A) and few off target edits using a gRNA homology-based approach. In cells differentiated at air–liquid-interface (ALI), 17% and 52% editing of CFTR G542X increased mRNA abundance. 52% editing alone or 17% and 52% editing of CFTR G542X plus treatment with CFTR modulators (VX-445/VX-661/VX-770; ETI/Trikafta/Kaftrio) increased epithelial CFTR protein expression, CFTR protein band C abundance, CFTR_172_ inhibitable anion transport, and changes in airway surface liquid height and pH in response to vasoactive intestinal peptide (VIP) stimulation. Epithelial scratch repair speed and directionality was also improved. These data provide proof-of-concept that ABE of G542X to G542R in human CF airway epithelial cells could provide a feasible therapy for this variant.

**Graphical abstract:**

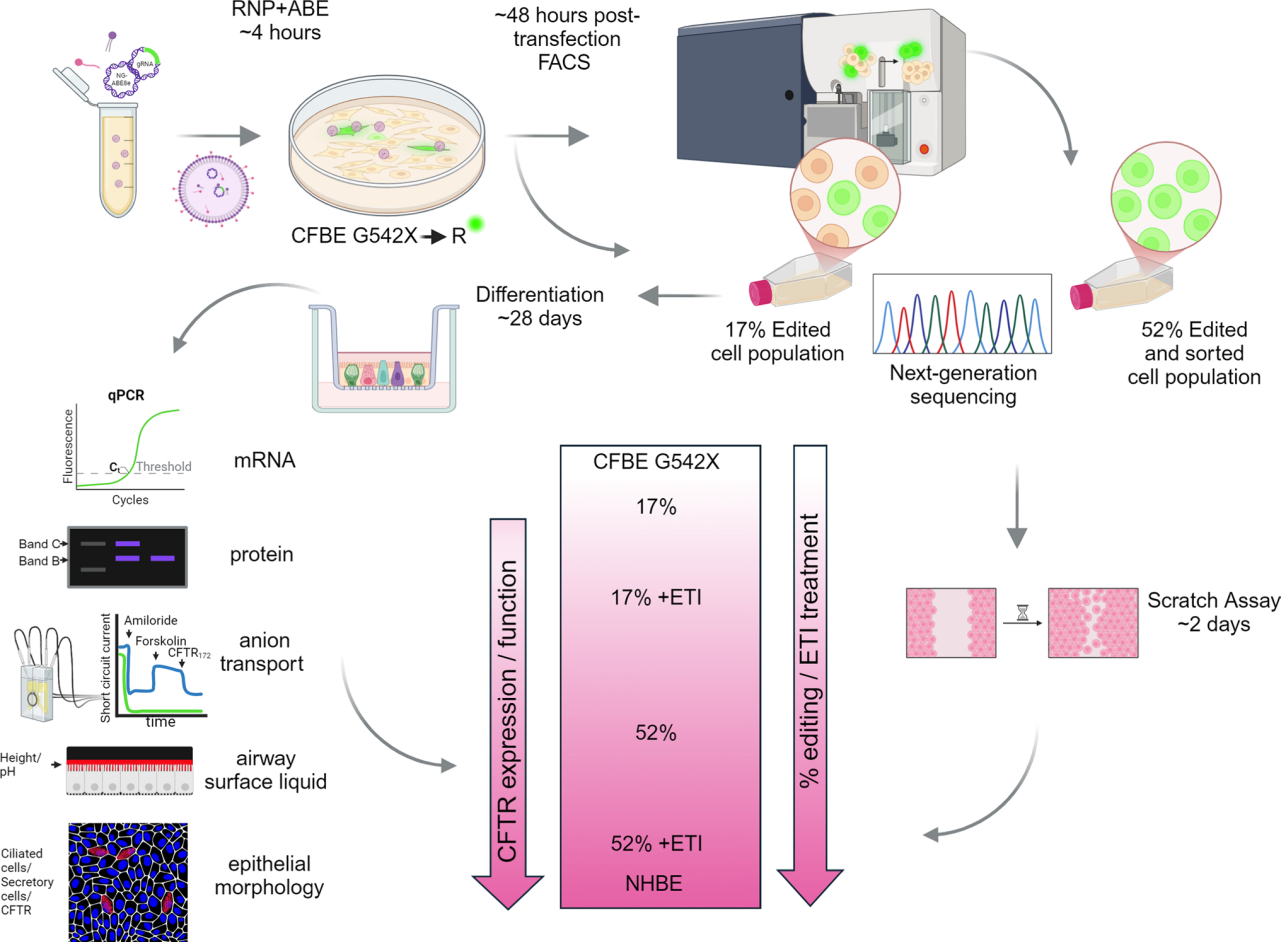

**Supplementary Information:**

The online version contains supplementary material available at 10.1007/s00018-025-05587-y.

## Introduction

Cystic fibrosis (CF) disease is a recessively inherited genetic disorder of the cystic fibrosis transmembrane conductance regulator (*CFTR*) gene which encodes an anion channel. Disease causing variations in the sequence of *CFTR* disrupt the formation of CFTR protein and lead to defective function of the channel. In epithelial tissues such as the lung, this is characterised by loss of surface hydration, mucostasis, infection and inflammation [1]. Variants that cause disease can be categorised into 6 major classes that include no transcription, no protein production, and dysfunctional protein [2, 3]. Increased understanding of the structure and function of CFTR has led to production of modulator drugs which potentiate (Vertex, VX-770) or correct (VX-661, VX-445, VX-809) CFTR protein [4]. These drugs in combination, e.g. Trikafta/Kaftrio (elexacaftor-tezacaftor-ivacaftor, ETI, VX445-VX661-VX770) have been transformative for many people with CF (pwCF), increasing quality of life by slowing the progressive lung disease, reducing the number of exacerbations and time spent in hospital [5]. However, modulator therapies are not applicable to ~ 10% of pwCF whose genetic variant prevents the production of CFTR protein. Finding other therapeutic options are therefore of key importance.

CF-causing single-nucleotide polymorphisms (SNPs) make up ~ 46% of total CF point mutations. Included in this group is the second most common CF-causing variant G542X (**G**GA > **T**GA) which results in the generation of a protein translation stop codon. The presence of the stop codon primarily leads to nonsense-mediated decay (NMD) of the mRNA resulting in lack of translation of CFTR protein (truncated or full length) and a severe loss of protein in airway epithelial cells [6]. Readthrough agents, including geneticin (G418), ELX-02 [7], PTC124 [8], SMG1i [9], SRI-41315 [10] alone, in combination or together with the use of CFTR potentiators/activators have been shown to promote G542X mRNA transcription and translation when expressed in 16HBE14o-, FRT cells, primary bronchial epithelial cells, patient derived organoids and in a humanised mouse model. Restoration of CFTR anion transport in these models ranges from ~ 3 to 15% of non-CF values. Also, the mechanism of action employed for premature stop codon suppression has the potential to cause a range of detrimental effects due to lack of specificity [11]. Therefore, there is precedent for utilising ABE to modify G542X so that treatment with already clinically effective modulators is possible.

The introduction of gene editing technologies, particularly CRISPR–Cas9, has heralded a new era in the potential treatment of genetic diseases like CF. However, despite huge advancements with CRISPR technologies already in the clinic, challenges such as delivery of gene therapies, editing efficiency, and the risk of unintended genetic alterations have, as yet, limited its clinical application [12, 13]. Prime-editing and base editing offer reduced risk of introducing double-strand breaks (DSBs) and the potential formation of insertions and deletions (indels) in the genomic DNA as seen in traditional CRISPR–Cas9 editing. Both offer promising alternatives for the correction of point mutations associated with CF [14–16]. Base editing enables the direct, irreversible conversion of adenine (A) to guanine (G) or cytidine (C) to thymidine (T) although other variants with wider capabilities are now emerging. By employing a catalytically inactive Cas9 nickase (nCas9) fused to an evolved *E. coli* tRNA adenosine deaminase enzyme (TadA), adenine base editors (ABE) target specific A nucleotides for conversion to G. Adenine base editing (ABE) has been used to correct the nonsense codon (TGA) associated with G542X to a missense codon **C**GA, encoding the variant G542R [17–19]. This variant enables mRNA transcription and protein production [20]. In this study, we explored the hypothesis that delivery of ABE using novel self-assembling receptor targeted lipid nanoparticles (RTN) and conversion of G542X to G542R could lead to a therapeutically functional treatment. We assessed editing efficiency of ABE delivered as plasmid DNA also encoding EGFP, by RTN to CFTR G542X homozygous airway epithelial cells. We used fluorescence activated cell sorting (FACS) to select EGFP expressing cells for enhancement of the edited cell population. Transfected cells were analysed for CFTR-specific functional changes in the presence and absence of ETI. Our objective was to provide proof-of-concept for RTN delivery of ABE as a feasible and safe method for correction of the G542X variant to restore CFTR-related physiological functions to the airway.

## Materials and methods

### Cell culture

Human BMI1 transduced non-CF (NHNE, S Hart, UCL, UK) and hTERT Bmi-1 transduced G542X CF airway epithelial cells (CFNE G542X, kind gift from S Randell, UNC, USA, [21]) p2, 8 were seeded on collagen (PureCol^®^) coated plasticware at a density of 3 × 10^5^ into 75 cm^2^ flasks with 15 ml PneumaCult™ Ex-Basal Culture media. Media was changed every 48 h. When flasks were 80–90% confluent, cells were detached with Trypsin/EDTA (0.25% solution) and cells were pelleted in 5 ml of PneumaCult™ Ex-Basal media at 500 rpm for 5 min. The cell pellet was re-suspended in 2 ml PneumaCult™ Ex-Basal media for counting before re-seeding.

### Air liquid interface (ALI) culture

NHNE, CFNE G542X and edited CFNE G542X were seeded onto collagen coated Snapwell culture inserts (cat no. 3801, Corning^®^) at a density of 0.5 × 10^6^ per 1.2 cm^2^ in 250 μl of PneumaCult™ Ex-Basal media, 1 ml of PneumaCult™ Ex-Basal media was added to the basolateral side. After 48 h the media was aspirated from both apical and basolateral compartments and basolateral side replaced with 1 ml PneumaCult™-ALI Medium which was refreshed every 48 h. Cells were cultured for a minimum of 21 days at ALI. Treatment with ETI was performed 24 h prior to functional assessment with 1 μM VX-445, 3 μM VX-661, and 1 μM VX-770.

### Transfection using RTN

Liposomes were a mixture of the cationic lipid 1,2-di-((Z)-tetradec-11-enyloxy)-N,N,N trimethylammonium propane chloride (DTDTMA or C14), and the neutral lipid Dioleoyl L-α phosphatidyl ethanolamine (DOPE), combined in a 1:1 molar ratio (Avanti Polar Lipids). Liposomes were prepared by mixing the lipids in ethanol and injecting into a NanoAssemblr microfluidics system at a flow rate of 12 ml/min (Precision Nanosystems), followed by sonication and dialysis against nuclease-free water overnight using Maxi GeBaFlex-tubing with an 8 kDa MWCO (Generon) and stored at 4 °C. Peptide E, K_16_GACSERSMNFCG (produced by AMS Bio) contained the SERSMNF receptor targeting motif identified by phage display biopanning on the human airway epithelial cell line (1HAEo-).

**Fig. 1:**
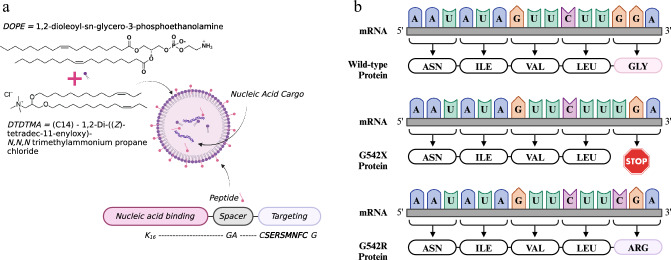
Adenine base editing. **a** Structure of the receptor targeted nanoparticle (RTN) complex showing formulation of the liposome complex (purple), targeting peptide structure (pink) and nucleic acid cargo (purple helices), **b** illustration showing the genomic changes in sequence from non-CF (glycine) to the G542X (stop) variant which causes premature termination of translation, to the edited G542R (arginine) sequence Created in BioRender.com

Plasmids were obtained from Addgene http://n2t.net/addgene. NG ABE8e Cas9 plasmid, Addgene plasmid #138491 [22] and EGFP gRNA_Cloning Vector BbsI, Addgene plasmid #128433. The sgRNA sequence was developed, optimised and kindly provided by L Nicosia and Prof P Harrison, University of Cork, Ireland [17]. Plasmids were prepared for transfection at a 1:1 molar ratio of ABE8e:gRNA vector. Receptor-targeted nanocomplexes (RTNs) (Fig. 1a) were prepared in water at a concentration of 4 μg / ml with respect to pDNA and in a 1:4:1 weight ratio of liposome: peptide: DNA and incubated at room temperature for 30 min to stabilise the RTNs prior to biophysical analysis. Size and zeta potential were measured in a Nano ZS Zetasizer (Malvern).

RTNs were prepared as above but in OptiMEM instead of water for transfections of ~ 1.4 × 10^6^ CFNE-G542X basal cells for 4 h. Plasmid uptake was visualised using confocal microscopy (Nikon A1R confocal microscope) and EGFP fluorescing cells quantified using ImageJ.

### Flow-cytometric cell sorting

24 h after transfection cells were washed with phosphate buffered saline (PBS). Trypsinisation (0.25% trypsin–EDTA) of CFNE expressing EGFP in cells which had taken up editing machinery yielded ~ 1.2 × 10^6^ cells from a single well (6-well plate). Cells were resuspended in 500 ml FACS buffer (Supplemental Table S1) and sorted by Fluorescence Activated Cell Sorting (FACS), Melody Cell Sorter, BD Biosciences. Singlets were discriminated from doublets using forward scatter (FSC) height vs FSC pulse area [23], and debris were excluded. Unsorted/sorted edited cells were seeded onto plastic (analysis of editing/scratch assay) or grown at ALI for subsequent functional analyses.

### Analysis of editing

Online tools (NCBI Blast, CRISPOR) were used to analyse genomic sequences with similar homology to the guide sequence. The top 5 exon spanning and top 5 intronic sites were selected based on the sequence similarity. Genomic DNA was extracted using QIAGEN DNeasy Blood & Tissue kit according to the manufacturer’s instructions. DNA concentration was determined using a NanoDrop 2000. Genomic sites of interest were amplified by PCR using appropriate primer pairs (Supplemental Table S2). PCR was carried out using high-fidelity Phusion DNA polymerase with thermocycling: 98 °C for 2 min, then 35 cycles of 98 °C for 10 s, 53 °C for 20s, and 72 °C for 20s, followed by a final extension of 72 °C for 1 min. PCR products were verified by comparison with DNA standards on a 1% agarose gel supplemented with SYBR-safe gel stain. PCR products were treated using ExoSAP-IT (ThermoFisher) for product purification. Samples were sent for next-generation sequencing (NGS) (GENEWIZ; https://www.genewiz.com). The sequence of G542X and G542R are shown in Fig. 1b.

### RT-qPCR

RNA was extracted from ALI cultures 28 days post plating using the RNeasy Mini Kit (QIAGEN) according to the manufacturer’s instructions. cDNA was generated using the Luna One-Step RT-qPCR kit (New England Biolabs) according to the manufacturer’s instructions alongside appropriate primers (Supplemental Table S2). Amplification was performed on mRNA extracted from fully differentiated ALI cultures four weeks post-editing using PCR protocol as described above and data shown as ΔΔCT = ΔCT(CFTR)-ΔCT(GAPDH).

### Western blot

ALI cultures were lysed in NP-40 Lysis Buffer, incubated on ice for 20 min then centrifuged at 15,000×*g* for 20 min. Protein concentration was determined by Pierce™ BCA Protein Assay Kit and 20 µg protein was denatured with LDS sample buffer and reducing agent (NuPAGE) at 65 °C for 15 min [24]. Samples were resolved on NuPAGE 4–12% Bis-Tris Protein Gels with mass standards 10–250 kDa (LI-COR). Proteins were transferred to Immobilon^®^-FL PVDF membrane (Millipore). Membranes were blocked in Odyssey^®^ Blocking Buffer (LI-COR), immunostained with anti-CFTR or anti-α Tubulin followed by IRDye^®^ 800CW Goat anti-Mouse IgG (LI-COR), visualised and quantified on an Odyssey IR imager (LI-COR) (Supplemental Tables S3 and S4).

### Ion transport

Transepithelial ion transport was measured in Ussing chambers using symmetrical bicarbonate buffered salt solution pH 7.4 (Supplemental Table S1), at 37 °C, bubbled with 21% O_2_, 5% CO_2_ and the following drugs were used: amiloride (apical, 100 μM) to inhibit the epithelial sodium channel (ENaC), forskolin (bilaterally, 10 μM) to activate CFTR and CFTR_inh_172 (apical, 10 μM) to inhibit CFTR as previously described [25] Data were analysed using LabChart 7 software (ADI Instruments). All drugs/chemicals were obtained from Sigma-Aldrich.

### Airway surface liquid height

Airway surface liquid (ASL) was labelled with 2 mg/ml Texas red-dextran (10 kDa) in PBS (20 μl) and left for 24 h to equilibrate. Perfluorocarbon (PFC) was added to the mucosal surface to prevent evaporation of the ASL, and the culture was placed on the stage of the confocal microscope over a serosal reservoir containing 80 ml of bicarbonate buffered salt solution (Supplemental Table S1). To determine the average height of the ASL, eight predetermined points on the culture were scanned using a Leica SP8 confocal microscope with a × 63/1.3 numerical aperture (NA) glycerol immersion lens in XZ-scanning mode as previously described [26].

### Measurement of ASL pH

ASL pH was measured using pH-sensitive pHrodo red dextran (10 mM), pH insensitive Alexa Fluor 647 dextran (10 mM) in bicarbonate buffered salt solution (20 μl) added to the apical surface of each transwell and incubated overnight in humidified air + 5% CO_2_ at 37 °C. Excitation/emission at 562/592nm and 650/668nm was measured the following day over a period of 8 h using a Tecan Spark. At 2 h 100 nM VIP was added basolaterally to activate CFTR. Apical pH was calculated as the fluorescence ratio pHrodo red dextran:Alexa Fluor 647 dextran less background fluorescence from non-labelled ALI cultures and results aligned to a standard curve generated from in situ controls of known pH 6.0–7.5) as previously described [27]

### Immunohistochemistry

ALI cultures were fixed in 4% paraformaldehyde (PFA) for 25 min then permeabilised with 0.2% Triton-X100 at room temperature. Samples were incubated with blocking buffer prior to incubation with primary antisera, prepared in PBS with 5% normal goat serum and 0.1% Triton-X100, both overnight at 4 °C. Samples were washed with PBS 3 × 30 min before counterstaining with appropriate secondary antisera (Supplementary Table S3), prepared in PBS with 0.1% Triton-X100, for 1 h at room temperature. Samples were washed with PBS 3 × 30 min prior to mounting with ProLong Gold Mountant with DAPI (Invitrogen). Images were taken using a Nikon A1R confocal microscope with a × 100/1.25 NA oil immersion lens.

### Scratch assay

Confluent basal airway epithelial cell cultures grown in PneumaCult™ Ex-Basal media were scratched mechanically using the AutoScratch tool. Images were taken every 25 min over 24 h using a LiveCyte 2 to assess collective migratory rate and cell directionality. Images were taken at three fixed points along the scratch edge for each well. Rate of wound closure was calculated using ImageJ software and cell directionality was tracked using LiveCyte Analyse and MATLAB software.

### Inflammatory markers

Media samples were collected from non-transfected or transfected cultures 24 h prior to transfection and post-transfection (0–72 h) RTN plus ABE DNA or RTN alone. Treatment with IL-1β was used as a positive control. Cytokines were measured using human IL-6 and IL-8 Quantikine ELISA kit (Bio-techne).

### Statistics and data analysis

Normally distributed data were analysed using ANOVA followed by Tukey’s test or unpaired t-test with Welch’s correction. Non-parametric equivalents (Mann–Whitney test, Kruskal–Wallis test with Dunn’s multiple comparisons test) were used when data were not normally distributed. Data are shown as individual points and/or mean ± standard deviation (SD). Significant differences are indicated with *p < 0.05; **p < 0.01 ***, p < 0.001, ****p < 0.0001. Data analyses were performed using GraphPad Prism v10.2.2 (GraphPad Software).

## Results

### RTN delivered ABE precisely edits G542X

**Fig. 2:**
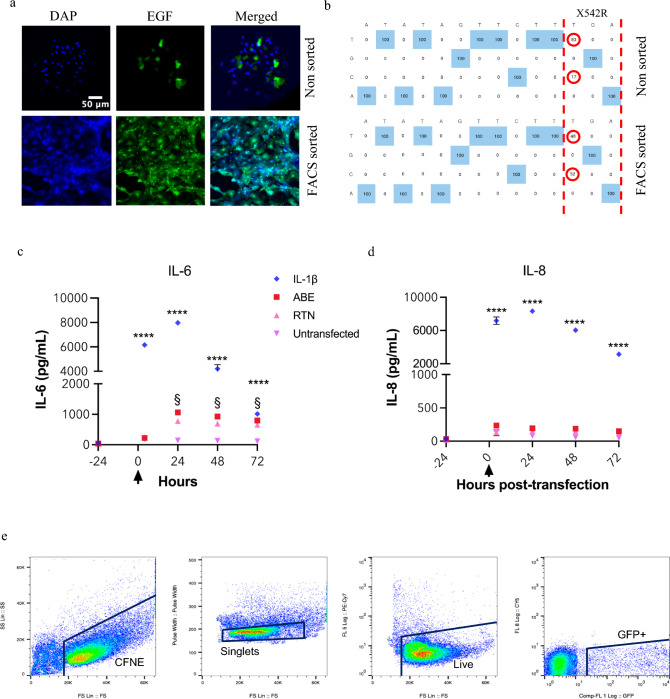
RTN delivered ABE8e/sgRNA specifically and efficiently edits CFNE G542X airway epithelial cells. **a** Representative fluorescent microscope images of basal cells transfected with NG-ABE8e Cas9/EGFP-sgRNA and expressing EGFP (green) before (upper panel) and after-fluorescent activated cell sorting (FACS) (lower panel) to select for EGFP expressing cells. Cell nuclei stained with DAPI (blue), cells expressing EGFP (green) and overlay. Scale bar 50 µm shown on top left image. **b** Next-generation sequencing of the G542X genomic region from edited and edited/FACS sorted CFNE G542X cells. Numbers circled in red show 17% and 52% allele correction of the correct base (T to C) in the G542X codon (highlighted with dashed red lines) and with no other alterations in the editing window as shown. **c** and **d** Measurement of IL6 and IL8, respectively, in the medium 24 h before treatment with IL-1β (positive control) or transfection with ABE8e/gRNA/RTN (ABE) or RTN alone (RTN) at 0 h (arrow), after 20 min and up to 72 h later. Untransfected/untreated cells (untransfected) are included as a negative control. Statistically different to untransfected cells ****p < 0.0001, statistically different to untransfected cells and treated cells ^§^p < 0.0001. **e** Gating scheme for airway epithelial cells and purity of cell population after FACS. Representative flow cytometry plots show CF airway epithelial (G542X) transfected GFP^+^ cells

RTN formulations carrying ABE plasmids in water were 122.7 ± 0.9 nm with a polydispersity index (PDI) of 0.546, and zeta potential difference of + 40.06 mV. RTNs formulated in the same way but carrying NG-ABE8e Cas9, EGFP and sgRNA were used to transfect CFNE G542X cells (Fig. 1a). Fluorescence microscopy indicated that there were 37 ± 4% EGFP expressing cells 24 h after transfection. A sample of this population was retained while another was subjected to FACS to obtain an enriched population of EGFP fluorescing cells (sorted cells; 99 ± 0.2% EGFP expression). Sanger sequencing of genomic DNA indicated that 17% of alleles in the unsorted cells and 52% of alleles in the sorted cells exhibited editing of the targeted A, resulting in a conversion of T to C on the coding strand (Fig. 2a, b). This indicated a transfection to allele editing ratio of ~ 2:1, respectively. No editing of adjacent As were observed. Analysis of the top 10 genomic regions exhibiting homology to the guide RNAs were subjected to Sanger sequencing and analysed for potential off target edits using CRISPOR. Of these, 7 showed no changes in sequence, one showed a change from A to G but with no effect on the amino acid codon, and two exhibited editing in a deep intronic region (Supplemental Figure S4). Further analysis over 3 months showed that the number of base edited alleles in the population was stable with editing remaining at 17% at P7, 19% at P15 and 20% at P21.

### RTN delivered ABE had little effect on IL-6 and IL-8 secretion

Transfection of *CFNE G542X* with RTN alone or RTN containing ABE induced a small elevation in IL-6 that was significantly lower than the response to IL-1β (p < 0.0001, n = 6). (Fig. 2c) Transfection of cells had no effect on secretion of IL-8 (Fig. 2d).

### RTN delivered ABE of CFNE G542X restored CFTR mRNA and protein abundance

**Fig. 3:**
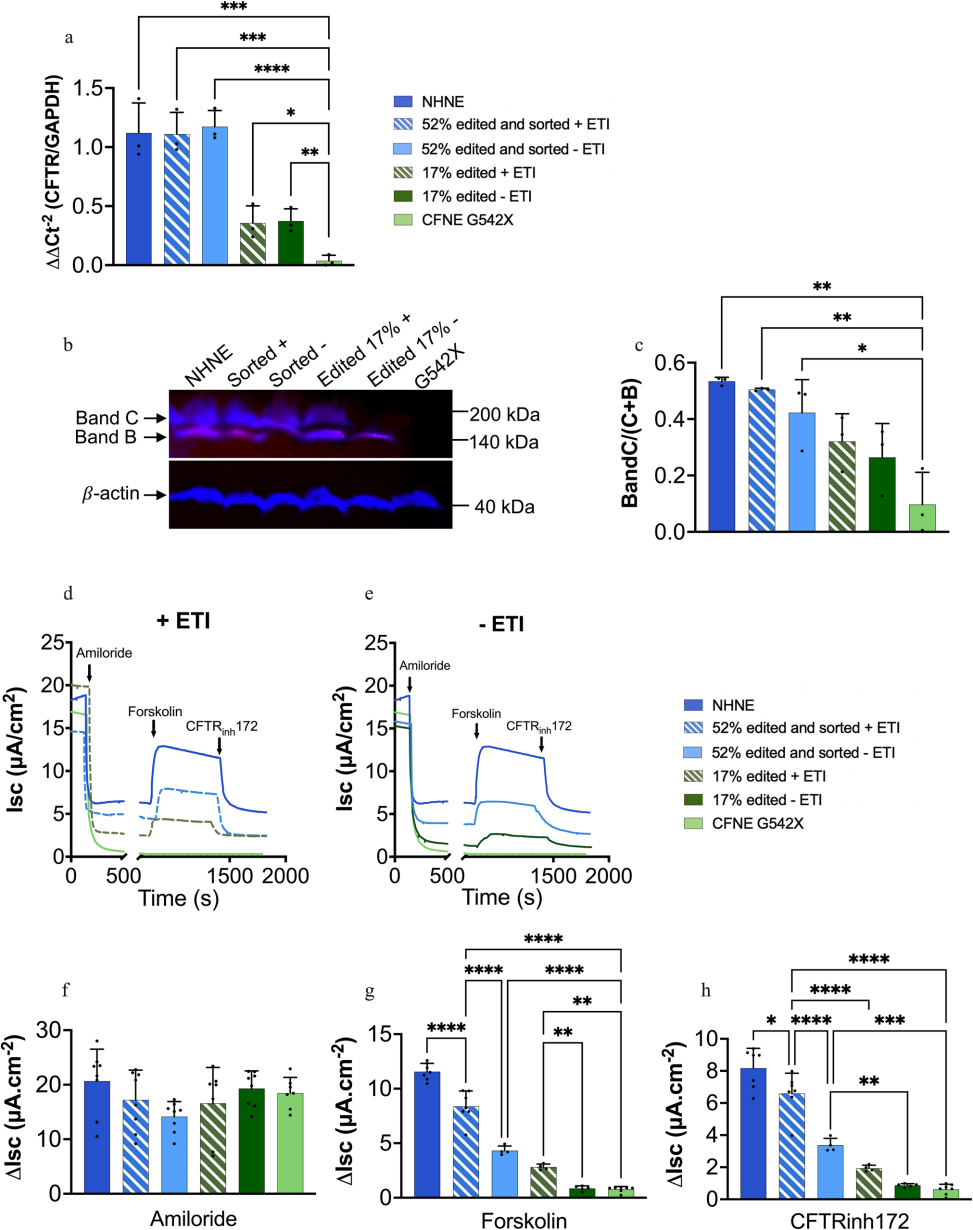
ABE of CFNE G542X restores CFTR expression, anion transport and response to modulators. **a** RT-qPCR of CFTR mRNA extracted from NHNE, unedited (CFNE G542X), edited (Unsorted 17%) and edited and FACs sorted (Sorted 52%) cells grown at ALI and treated with vehicle (− ETI) or ETI (+ ETI). Data were calculated as 2^−ΔΔCt^ RNA quantification of CFTR normalized to GAPDH (n = 3). **b** Representative image of western blot showing CFTR protein bands B (immature/intracellular) and C (mature/membrane CFTR) and β-actin (loading control) from samples as described in **a**. **c** Quantification of CFTR bands C/B + C as shown in b. (n = 3) using OdysseyCLx fluorescence data. Representative short circuit current (I_sc_) traces from NHNE, CFBE G542X, unsorted 17% and sorted 52% cells after addition of amiloride (10 mM, apical), forskolin (10 mM bilateral), CFTR inhibitor172 (10 mM, apical) as indicated. **d** I_sc_ traces from unsorted 17% and sorted 52% cells that had been treated with ETI (+ ETI) or with vehicle (− ETI). **e** Untreated NHNE and CFBE G542X are the same on both graphs for reference. Change in I_sc_ (ΔI_sc_) in response to **f** amiloride, **g** forskolin and **h** CFTR inhibitor172 (n = 6). Data are shown as mean ± SD. Significantly different as shown, *p < 0.05, **p < 0.01, ***p < 0.001, ****p < 0.0001. The key to graphs and traces is also shown to the middle right of the figure

In edited CFNE G542X cells grown at air–liquid-interface (ALI) containing 17% G542R alleles, mRNA abundance was 0.37 ± 0.10 (2^−∆∆Ct^, n = 3) compared to non-edited cells. In the edited and sorted cells (52% G542R alleles) mRNA abundance was restored to levels observed in non-CF NHNE (1.20 ± 0.25 and 1.11 ± 0.18, respectively, n = 3). As expected, pre-treatment with ETI had no effect on mRNA abundance (n = 3) (Fig. 3a).

No full length or truncated CFTR protein was observed in CFNE G542X by western blot (Fig. 3b and Supplementary Figure S6a). Immature CFTR protein band B was predominant in 17% edited cells with little of the glycosylated CFTR protein band C. In edited sorted cells band B and C were more abundant and the C/B + C ratio was increased (p < 0.001, n = 3). Treatment with ETI further increased CFTR band C abundance and the C/ B + C ratio in 17% edited and edited sorted cells (p < 0.05, n = 3) respectively. The C/ B + C ratio was similar in edited sorted cells and NHNE) Fig. 3b, c and supplementary Figure S6b.

### RTN delivered ABE of CFNE G542X restored CFTR mediated anion transport, ASL height and pH.

There was no difference in transepithelial electrical resistance (TEER, Supplementary Table S5) or amiloride-sensitive I_sc_ between edited and unedited CFNE G542X cells grown at ALI (n = 6) (Fig. 3d, e). In 17% edited cells, forskolin-stimulated I_sc_ and CFTR_172_-inhibitable I_sc_ were not different to non-edited cells. However, treatment with ETI significantly increased I_sc_ to approximately 24% of non-CF levels (p < 0.05, n = 4). In edited and sorted cells, forskolin-stimulated and CFTR_172_-inhibitable I_sc_ was increased to approximately 41% of non-CF levels and was indistinguishable from non-CFNE levels after treatment with ETI (p < 0.001, n = 6 respectively) (Fig. 3f, g, h and Supplementary Figure S7a, b).

**Fig. 4:**
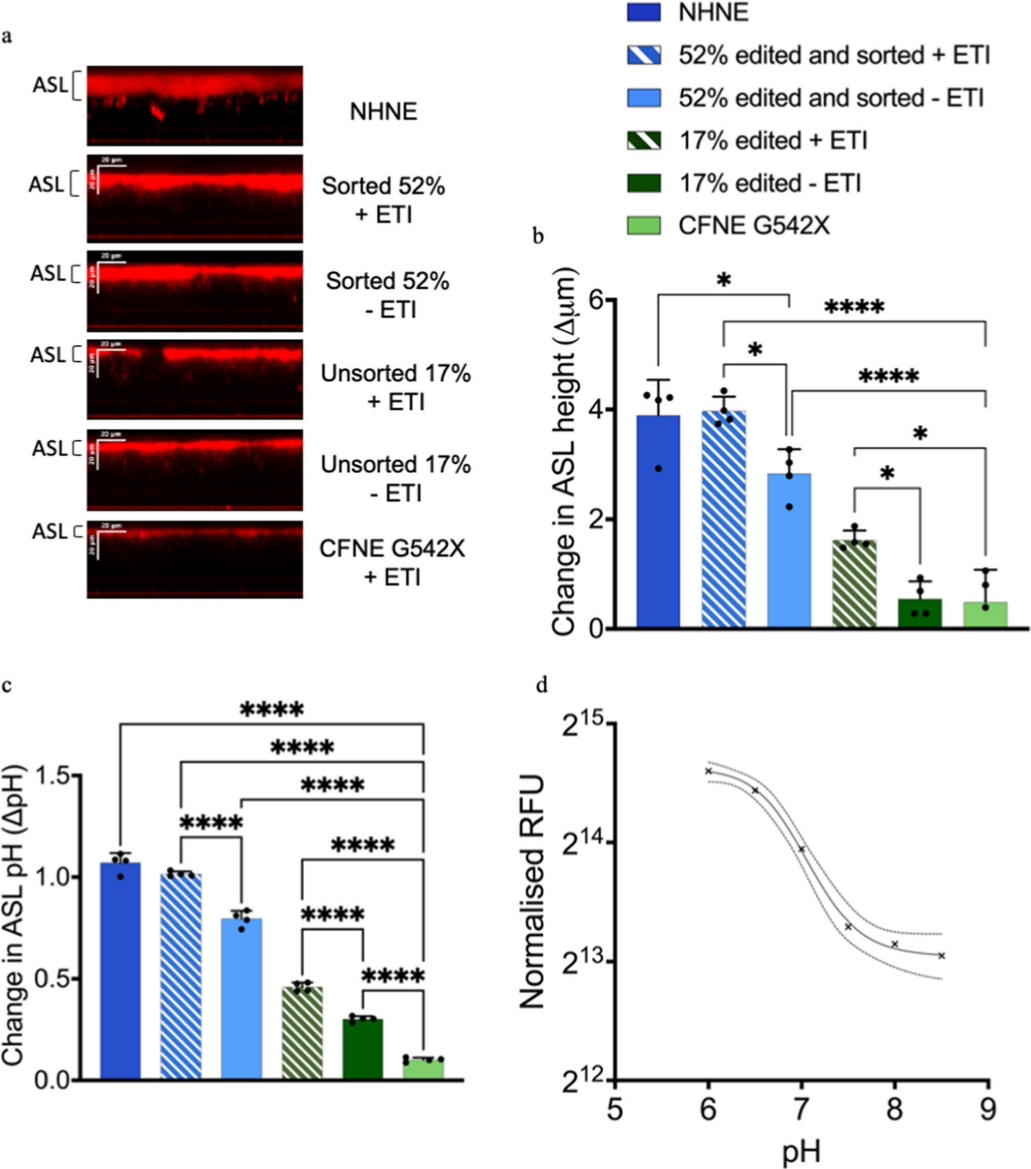
ABE of CFNE G542X restores ASL height and pH. **a** Representative confocal XZ images showing depth of ASL (red) overlying NHNE, CFBE G542X, unsorted 17% and sorted 52% grown at ALI in response to stimulation with vasoactive intestinal peptide (VIP, basolateral), after treatment with vehicle (− ETI) or ETI (+ ETI) as indicated. **b** Change in ASL height in response to VIP quantified using ImageJ. **c** Change in ASL pH, interpolated from **d**. standard curve used to calculate ASL pH (see “Methods”). Data are shown as mean ± SD. Significantly different as shown, *p < 0.05, ****p < 0.0001, n = 6

Elevation of cAMP with vasoactive intestinal peptide (VIP) applied to the serosal surface increased ASL height in a similar pattern to that observed with CFTR mediated anion transport described above (n = 8 replicates per well from n = 2 independent experiments). There was no change in ASL height detected in CFNE G542X cells or 17% edited cells, but there was an increase in ASL height (to ~ 75% of non-CF) in edited and sorted cells (p < 0.0001). The increase in ASL height was further enhanced by ETI treatment (to ~ 30% of non-CF ASL height) in 17% edited and was indistinguishable from the response of non-CF cells in sorted and edited cells (p < 0.05 and p < 0.0001 respectively) (Fig. 4a, b). We were unable to determine differences in baseline ASL height due to the resolution of the experimental technique.

The change in ASL pH on administration of serosal VIP, was consistent with increased CFTR—mediated HCO_3_^−^ secretion from CFNE G542X 17% edited and sorted edited cells. ∆pH was enhanced by treatment with ETI (all p < 0.0001, n = 6). Interestingly the increase in ∆pH between non-edited CFNE G542X and 17% edited cells we did not observe when measuring anion transport or ASL height. This could be associated with differing sensitivities of the two methodologies or that G542R CFTR somehow permits more efficient HCO_3_^−^ secretion (Fig. 4c). No changes in pH could be determined at baseline because exposure to environmental air during the measurement process induces changes in pH that mask any subtle differences at baseline.

### RTN delivered ABE of CFNE G542X restored migration and directionality of wound repair

**Fig. 5:**
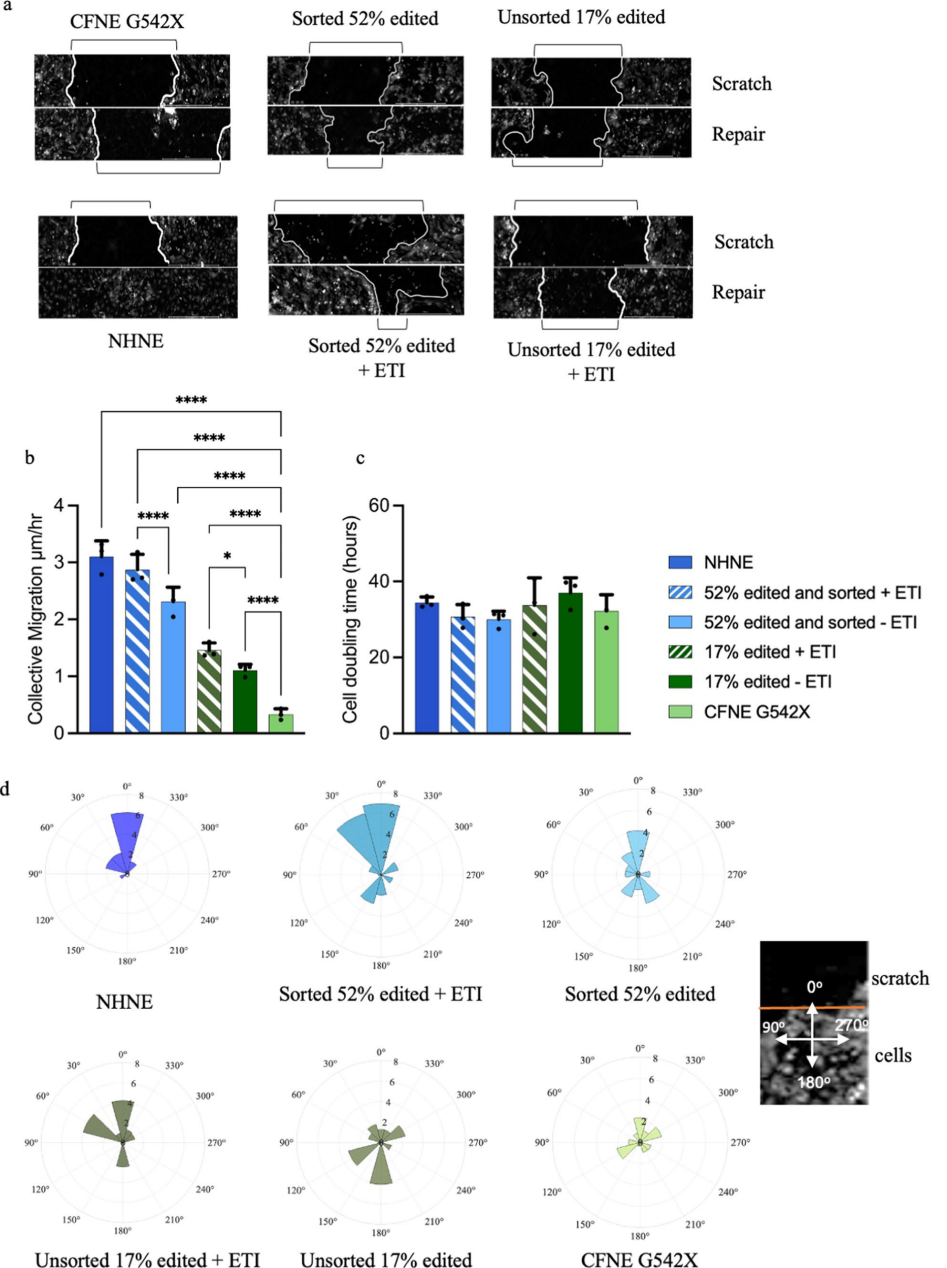
ABE of CFNE G542X improves scratch repair. **a** Representative LiveCyte 2 microscope images of NHNE, CFBE G542X, unsorted 17% and sorted 52% basal cells before and after treatment with vehicle (− ETI) or ETI (+ ETI) showing scratch and repair over a period of 35 h. Scratches before and after are indicated by black markers. **b** Collective migration in mm/hour and **c** cell doubling time (hours) quantified from LiveCyte 2 images obtained in a. shown as mean ± SD from n = 6 replicates from 2 independent experiments. **d** Directionality cones of cell migration during wound scratch repair. Scratches are orientated to 0° as shown in the image to the left. Cones facing 0° indicate cell migration towards the direction of the scratch, and size of cone reflects number of cells migrating in a similar direction

In the scratch assay, collective migration and repair of the scratched area by encroaching cells was delayed in CFNE G542X compared to 17% or 52% sorted edited CFNE G542X and NHNE cells (p < 0.0001, n = 6 replicates from 3 independent experiments). Treatment of 17% and 52% edited CFNE G542X cells with ETI further increased collective migration (p < 0.05 and p < 0.0001, respectively) (Fig. 5a, b and Supplemental videos S5). There was no difference in cell doubling time between edited and non-edited cells or with ETI treatment (Fig. 5c). Analysis of individual cells at the repairing edge of the scratch site showed a loss of directionality in CFNE G542X compared to NHNE cells. Directionality was improved in edited cells and enhanced with ETI treatment (Fig. 5d).

### RTN delivered ABE of CFNE G542X grown at ALI exhibit tight junctions, ciliated and secretory epithelial cell types.

**Fig. 6:**
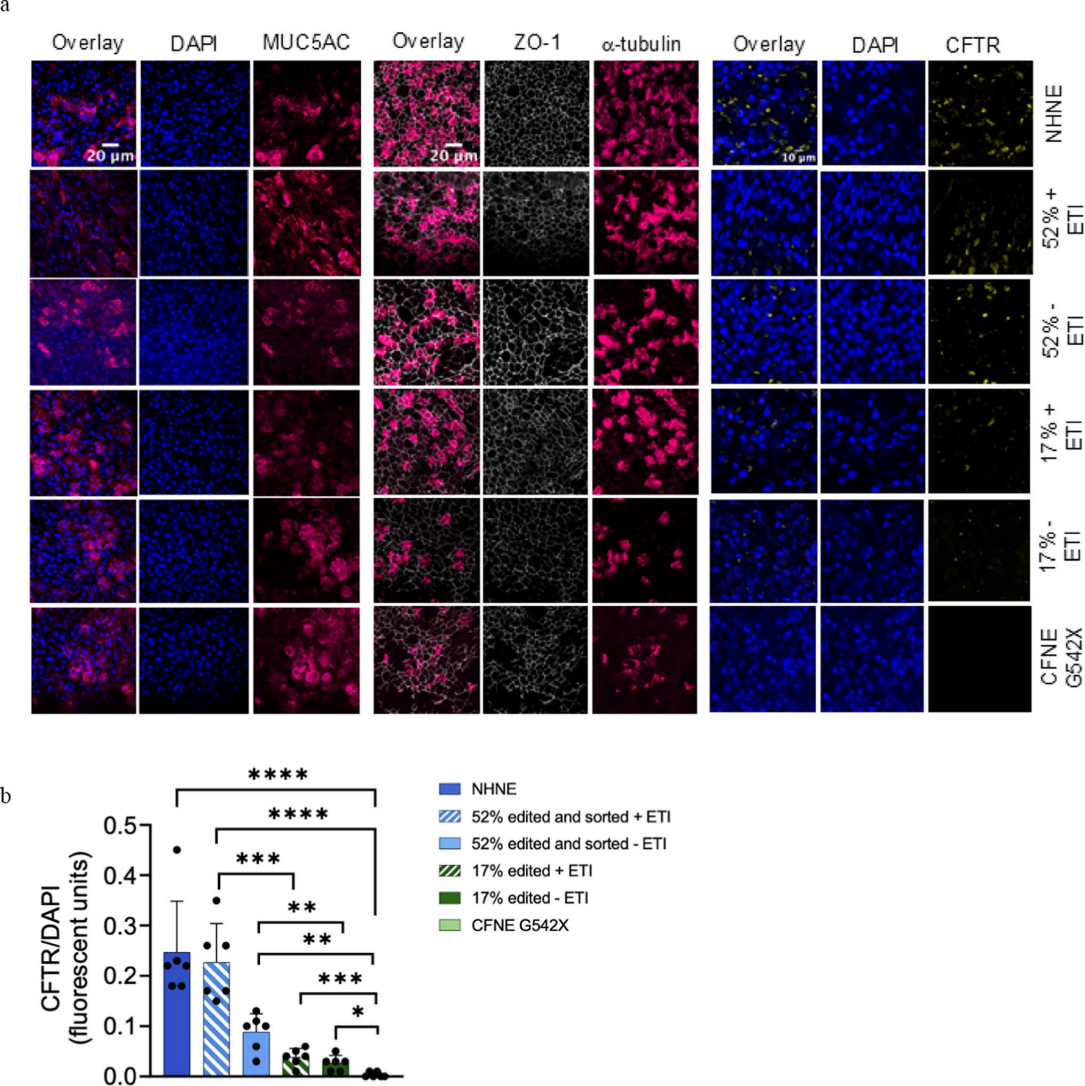
CFNE G542X cells subjected to ABE differentiate into ciliated and secretory cells. **a** Representative confocal microscopy images of NHNE, CFNE G542X, and edited cells grown at ALI with (+) and without (–) treatment with ETI and immunostained with MUC5AC (secretory cells red) and DAPI (nuclei, blue); α-tubulin (cilia, pink red) and Zona Occludens (ZO-1, tight junctions, cyan/white); CFTR (yellow) and DAPI (nuclei, blue). Overlays are shown to the left and a scale bar is shown to the lower right of the first image of each set (MUC5AC/α-tubulin, 20 μm, × 60 magnification lens, CFTR 10 μm, × 100 magnification lens). **b** Quantification of CFTR immunofluorescence (CFTR/DAPI) taken from three randomly selected fields of view from two independent transwells. Data are shown as mean ± SD. Significantly different as shown, *p < 0.05, **p < 0.01, ***p < 0.001, ****p < 0.0001, n = 3

After editing and when cultured at ALI, edited cells exhibited the presence of ciliated cells, secretory cells and the presence of tight junction protein ZO-1 that was similar to NHNE and CFNE G542X, indicative of a differentiated airway epithelium (Fig. 6). There was no significant change in ciliated/secretory cells in CFNE G542X or edited cells. However, immunostaining for CFTR was increased in edited cells with or without treatment with ETI in a similar pattern to that observed in the western blots (Figs. 3b, c 6a, b) and Supplementary Figure S8.

## Discussion

We present data that demonstrate successful RTN delivered ABE of the CFTR variant G542X in human airway epithelial cells. The RTN formulation comprises the peptide K_16_GACSERSMNFCG in which, K16 mediates mRNA binding and SERSMNF is a peptide ligand for epithelial receptors [28]. The synergistic interaction of the lipid and peptide components enables efficient packaging and protection of the nucleic acids. The peptide also targets transfection to the epithelial cell, while the lipids mediate efficient endosomal release.

We obtained 17% edited alleles with a single transfection of basal Bmi-1 transduced G542X CFNE with no editing of neighbouring As in the editing window. Enriching the edited cell population by utilising EGFP co-transfected with sgRNAs resulted in 52% edited alleles. As the cells were homozygous for G542X, assuming there is equal chance for one or two alleles to be edited per cell, this would potentially mean that at least 26% and 78% cells carried at least one G542R allele respectively. The editing values were similar to that using a shuttle peptide to deliver ABE8e Cas9 to CFF 16HBEge cells (20%) [29] and the 16% editing of F508del HBEC using lung selective organ targeting (SORT) lipid nanoparticles and homology directed repair (HDR) [30]. Using a guide RNA homology approach we also found only 3 off target edits at lower editing %, two of which were in introns and one coding region that did not result in an amino acid change. We suggest that ABE delivered and analysed in this way is more precise than other reported methods, but other approaches would be required to confirm this. For example, using EndoV- sequencing, Liang and colleagues found that ABE gave rise to an average of 8 (2–19) off target effects compared to an average of 161 (7–320) with HDR [31, 32]. We found a 2:1 transfection to allele editing efficiency, although in terms of edited cells this likely much higher. Unlike HDR, ABE with nCas9 is not dependent on the cell cycle increasing the efficiency of ABE compared to other methodologies [33]. Going forward, transient expression of EGFP with the sgRNA will be a useful tool to optimise ABE for different cell types and differentiation status in vitro and in vivo. In addition, the substitution of EGFP with other translational cell markers such as truncated tCD19, to produce a pool of highly edited basal cells will be useful for re-engraftment studies [34].

Reduced TEER, changes in tight junction localisation and decreased ciliated/goblet cell ratio have been reported in CF compared to non-CF cells [35, 36] although there remains discussion whether these changes are CFTR dependent [36]. The edited basal cells differentiated into key airway cell types (ciliated and mucous secreting) characteristic of a resistive airway epithelium. However, we did not observe any differences in TEER, ZO-1 staining or the ciliated/goblet cell ratio in CFNE G542X and edited cultures. This finding may reflect the characteristics of these particular cells and/or may be associated with the in vitro culture conditions. Consistent with previous studies, CFTR mRNA and protein in G542X CFNE grown at ALI was not detectable [37]. Editing of G542X to G542R in these cells increased mRNA abundance and full-length protein translation, although there was little mature glycosylated CFTR band C in 17% edited cells compared to 52% edited cells and NHNE cells expressing CFTR. Anion transport increased with editing efficiency, and the abundance of CFTR band C. Although anion transport values were lower than predicted for correction to non-variant CFTR, the trend was similar to what we previously observed when increasing NHBE in mixed epithelial populations of NHBE plus CFBE F508del, which mimicked an edited vs non edited cell population [25]. The edited cell populations responded robustly to ETI which elevated CFTR protein C abundance, the C/C + B ratio and all other physiological processes we measured. G542R CFTR was reported to exhibit 40–70% of non-variant CFTR activity that was associated with a reduced band C:B ratio when expressed in HEK293T cells. Treatment with the CFTR corrector VX809 increased G542R protein abundance and the C:B ratio in these cells. VX809 increased G542R CFTR mediated Cl^−^ transport in FRT cells and treatment with the CFTR activator VX770 further increased I_sc_ [20, 38]. Thus, we propose that in untreated G542X edited cells, anion transport activity is determined by the number of edited alleles which drives abundance of CFTR protein, the band C/C + B ratio (which is reduced compared to non-variant CFTR) and channel activity. Treatment with ETI (correctors VX445 and VX661) stabilised the immature form of the protein and facilitated its translocation to the membrane increasing CFTR abundance and the C/C + B ratio. ETI (potentiator VX-770) activated the channel at the cell surface, together resulting in increased function.

G542X CFNE cells exhibited defective directionality and speed of wound repair which was partially restored by editing and treatment with ETI. Proliferation of basal cells can be donor dependent [25] but wound repair in scratch assay was shown to be slower in CF (CuFi-1) as opposed to non-CF (Nuli-1) cell lines, in primary bronchial epithelial cells from pwCF compared to non-CF (all homozygous for CFTR F508del) and to be associated with a migratory defect that was CFTR dependent [39]. Consistent with our results and the role of active CFTR in wound repair, ETI improved wound healing in CFBE14o- expressing the CFTR F508del mutation [40] and the use of correctors (VRT-325, VX-809 and VX-770) improved repair in airway epithelial cells from pwCF homozygous for CFTR with F508del and with F508del heterozygous with N1303K or I507del [39, 41]. While we recognise that wound repair in differentiated epithelia is more complex, these data indicate that damage repair in CF lungs could be improved by gene editing of class 1 variants to modulator responsive variants. While we do not yet have long term data, over a course of 21 basal cell passages, we saw a small increase in the number of edited alleles indicating that edited cells may also have a selection advantage. Even if this is not the case, others have found that gene editing of basal cells in vivo remained stable in the murine lung over 660 days [30].

Our data indicate that 17% allele correction by ABE plus ETI gave ~ 30% restoration of non-variant CFTR function and drove positive changes to key airway physiological processes. This value was higher than the minimum 10% residual function suggested to prevent respiratory decline and a potentially achievable target for inhaled therapies [42]. Sorted and edited cells after treatment with ETI exhibited function comparable to non-variant CFTR. Obtaining editing efficiencies of 52% with a single RTN transfection and without cell selection is challenging. Recently, an ABE editing efficiency of 45% was described in lung basal cells from R553X mice after repeated intravascular dosing of SORT delivered via the vascular route [30]. Our finding that RTN delivery of ABE induced low inflammatory responses would make repeated delivery a viable option. We have successfully repeat delivered RTN gene editing material to other CF cells in vitro [43]. Formulations have been nebulised to the conducting airways of the murine and porcine lung, with efficient levels of gene expression [44]. We have also shown in vivo lung delivery of pDNA, minicircle and siRNA [28, 44–50]. Delivery of the ABE as mRNA with synthetic sgRNA rather than plasmid DNA could further increase editing [51].

In summary, we have provided proof of concept that RTN delivered ABE of G542X CF airway epithelial basal cells creates a precisely edited and functional CFTR G542R protein. When edited cells were grown at ALI, CFTR G542R was responsive to treatment with the triple modulator therapy (ETI/Trikafta/Kaftrio) and restored key CFTR mediated physiological processes to the airway epithelium. Further work is now planned to optimise luminal RTN delivery of ABE as mRNA to differentiated G542X epithelial cells in vitro and in vivo.

## Supplementary Information

Below is the link to the electronic supplementary material.

Supplementary file1 (DOCX 448 KB)

## Data Availability

The experimental data that support the findings of this study are available in Figshare with the identifier 10.24376/rd.sgul.26927992.
