# Correction: Adenine base editing of CFTR using receptor targeted nanoparticles restores function to G542X cystic fibrosis airway epithelial cells

**DOI:** 10.1007/s00018-025-05907-2

**Published:** 2025-09-27

**Authors:** Isabelle Rose, Miriam Greenwood, Matthew Biggart, Natalie Baumlin, Robert Tarran, Stephen L. Hart, Deborah L. Baines

**Affiliations:** 1 Institute of Infection and Immunity, School of Health and Medical Sciences, City St George’s, University of London, London, SW17 0RE UK; 2 Department of Genetics and Genomic Medicine, UCL Great Ormond Street Institute of Child Health, London, WC1N 1EH UK; 3 Division of Genetic, Environmental and Inhalational Disease, University of Kansas Medical Center, Kansas City, KS 66160 USA; 4 Department of Internal Medicine, University of Kansas Medical Center, Kansas City, KS 66160 USA

**Correction: Cellular and Molecular Life Sciences (2025) 82:144**

10.1007/s00018-025-05587-y

In the original published article, RNP (ribonucleoprotein) in graphical abstract should read RTN (receptor targeted nanoparticle).

We would also like to clarify the vectors and constructs used in the study. Plasmid vectors were obtained from Addgene http://n2t.net/addgene. These were NG-ABE8e Cas9 plasmid (plasmid #138491) [22] and gRNA_Cloning Vector BbsI (plasmid #128433). CMV-EGFP was PCR amplified from pCMV-GFP (plasmid #11153) for directional cloning into the gRNA cloning vector using the EcoRI/XhoI restriction sites. The sgRNA sequence was cloned between the two BbsI restriction sites downstream of the U6 promoter. Thus, the plasmids used throughout the study were NG-ABE8e Cas9 and EGFP sgRNA combined at a 1:1 molar ratio. RTNs were prepared using a 1:4:1 weight ratio of liposome:peptide:DNA as outlined in the manuscript.

