# Correction: Adenine base editing of CFTR using receptor targeted nanoparticles restores function to G542X cystic fibrosis airway epithelial cells

**DOI:** 10.1007/s00018-025-06050-8

**Published:** 2026-01-30

**Authors:** Isabelle Rose, Miriam Greenwood, Matthew Biggart, Natalie Baumlin, Robert Tarran, Stephen L. Hart, Deborah L. Baines

**Affiliations:** 1 Institute of Infection and Immunity, School of Health and Medical Sciences, City St George’s, University of London, London, SW17 0RE UK; 2 Department of Genetics and Genomic Medicine, UCL Great Ormond Street Institute of Child Health, London, WC1N 1EH UK; 3 Division of Genetic, Environmental and Inhalational Disease, University of Kansas Medical Center, Kansas City, KS 66160 USA; 4 Department of Internal Medicine, University of Kansas Medical Center, Kansas City, KS 66160 USA

**Correction: Cellular and Molecular Life Sciences (2025) 82:144**

10.1007/s00018-025-05587-y

"In this article, certain instances of the term ‘nasal epithelial cells’ should be replaced with ‘airway epithelial cells’ in the body text."

The original article has been corrected.

