# Supplementary material for: Adenine base editing of CFTR using receptor targeted nanoparticles restores function to G542X cystic fibrosis airway epithelial cells
Source: Cell Mol Life Sci. 2025 Apr 7;82(1):144. doi: 10.1007/s00018-025-05587-y (PMC11977081; doi:10.1007/s00018-025-05587-y)
Supplement: Supplementary file 1 — Supplementary file1 (DOCX 448 KB) [file 18_2025_5587_MOESM1_ESM.docx]

Supplementary Information

Supplementary Table 1 (S1): Buffer composition

| Solution | Composition |
| --- | --- |
| Bicarbonate buffered salt solution | 117 mM NaCl, 2.5 mM CaCl2, 4.7 mM KCl, 1.2 mM MgSO4, 25 mM NaHCO3, 1.2 mM KH2PO4, 11 mM D-glucose (pH 7.4) |
| NP-40 Lysis Buffer | 25 mM Tris-HCl pH 7.4, 150 mM NaCl, 1 mM EDTA, 1% NP-40, 5% Glycerol, 10 ml cOmplete protease inhibitor cocktail. |
| FACS Buffer | PBS, 0.05% EDTA |

Table S1: Buffer compositions used for anion transport measurements and ASL serosal bath (bicarbonate buffered solution), protein extraction (NP-40 lysis buffer), and cell resuspension for FACS (FACS buffer)

Supplementary Table 2 (S2): Primers used for RT-qPCR and NGS

| Name | Forward primer sequence 5’ > 3’ | Reverse primer sequence 5’ > 3’ |
| --- | --- | --- |
| CFTR (RT-qPCR) | GGAGAGCATACCAGCAGTGACT | TTCCAAGGAGCCACAGCACAAC |
| GAPDH (RT-qPCR) | GTCTCCTCTGACTTCAACAGCG | ACCACCCTGTTGCTGTAGCCAA |
| CFTR Exon 12 (NGS) | TGCCTTTCAAATTCAGATTGAGCA | ACCAAGATACGGGCACAGAT |
| ADAMST1 | TGAAAGTGATCTACCTCAAAGA | TACTCATTTCCTGACCAAGGCTGC |
| DMTF1 | GCAGCTTTTTTCCCCTACAGGGT | CCTCAGAGATAAGCTTGCTGCC |
| IRAG1 | CACTTAATGCATCCCCAGCACAC | CCCTGCATATGAACTCATGGGGA |
| PCAT2 | GCCACCCAGACTGTGATATTTTGC | GGAGATAATCAGATCATTTCGTCA |
| STARD13 | GCCATTAGTTGATTTATTTAATGA | GCAGTCATCCATGTCCTCTTGTTC |
| Off-target intron 1 | CTCTTATAAGTCTTAGTTTCTGGT | CCCAGGAGAAAAGGACTGTGC |
| Off-target intron 2 | CTCTTAAATGTGAGCAGTTTTTAA | GGGTACAGTTGCTCCAAAGTGAC |
| Off-target intron 3 | GAATGAGTCTTGGTGCTTGGCAAC | CAGCCCTGCTGACACCTTGA |
| Off-target intron 4 | CTCTTATAAGTCTTAGTTTCTG | CCCAGGAGAAAAGGACTGTGC |
| Off-target intron 5 | CTCTTATAAGTCTTAGTTTCTGGT | CCCAGGAGAAAAGGACTGTGC |

Table S2: Primer sequences (forward and reverse) used for PCR.

Supplementary Table 3 (S3): Antisera and fluorophores

| Name | Species | Fluorophore | Dilution | Cat no. | Supplier |
| --- | --- | --- | --- | --- | --- |
| Anti-a-tubulin | Rabbit | - | 1:5000 | 500-6874 | Sigma-Aldrich |
| Anti-CFTR 596 | Mouse | - | 1:2000 | A4 | CFF Therapeutics |
| Anti-CFTR 450 | Mouse | - | 1:500 |  | CFF Therapeutics |
| Anti-ZO-1 | Goat | - | 1:2000 | PA5-19090 | Sigma-Aldrich |
| Anti-MUC5AC | Mouse | - | 1:2000 | sc33667 | SantaCruz Bio |
| Anti-Mouse IgG | Donkey | Alexa-fluor 594 | 1:1000 | A32744 | Thermo-Fisher |
| Anti-Rabbit IgG | Donkey | Alexa-fluor 555 | 1:1000 | 711-545-152 | Thermo-Fisher |
| Anti-Goat IgG | Donkey | Alexa-fluor 488 | 1:1000 | A32816 | Jackson Immuno |
| DAPI | - | Ex/Em 340/488 | 1:200 | D9542 | Sigma-Aldrich |

Table S3: Antibodies and fluorophores used for immunostaining and western blot.

Supplementary Table 4 (S4): Off-target analyses

| Gene/Intron | Result | Editing (%) |
| --- | --- | --- |
| ADAMST6 | GCCATGTCAAAGAACTATAT -- hit ACCTTCTCAAAGAACTATAT -- query | 4 |
| Off-target intron 1 | GGTTTCTCAAAGAACTATAT -- hit ACCTTCTCAAAGAACTATAT -- query | 0 |
| Off-target intron 2 | ACCTTTTACAAGAACTATAT -- hit ACCTTCTCAAAGAACTATAT -- query | 0 |
| Off-target intron 3 | TCCTTCCAAAGAACTATAT -- hit ACCTTCCAAAGAACTATAT -- query | 3 |
| Off-target intron 4 | GTTTCTCAAAGAACTATAT -- hit CCTTCTCAAAGAACTATAT -- query | 8 |
| Off-target intron 5 | GTTTCTCAAAGAACTATAT -- hit ACTTCTCAAAGAACTATAT -- query | 0 |
| DMTF1 | TTCTCTCAAAGAACTATAT -- hit ACCTCTCAAAGAACTATAT -- query | 0 |
| PCAT2 | ATCTTTTAAAGAACTATAT -- hit ACCTTTCAAAGAACTATAT -- query | 0 |
| IRAG1 | CCCTTCTCAGGAACTATAT -- hit ACCTTCTCAAGAACTATAT -- query | 0 |
| STARD13 | ACATTCCCAAAAACTATAT -- hit ACCTTCTCAAAAACTATAT -- query | 0 |

Table S4: Potential off target editing sites identified using CRISPOR. Hit refers to the sequence at the potential off target editing site and query refers to the sequence used to search for similar homology. Editing % refers to the % of alleles changed from A to G and the specific base edited is in red and underlined. For ADAMST6 the base change does not change the amino acid (serine).

Supplementary Table 5 (S5): Transepithelial Electrical Resistance

| NHNE | 52% + ETI | 52% - ETI | 17% + ETI | 17% -ETI | CFNE G542X |
| --- | --- | --- | --- | --- | --- |
| 868.8±68.02 | 845.3±66.29 | 737.1±107.72 | 716.5±83.32 | 731.5±72.09 | 757.3±66.85 |

Figure S5: TEER values taken prior to functional analyses. TEER (W/cm2) was measured using the EVOM2 voltmeter on the 28^th^ day of air-liquid interface cell culture. Values shown as mean ± SD (n = 6). There was no significant difference in TEER between the samples.

Supplementary Figure S6

a.


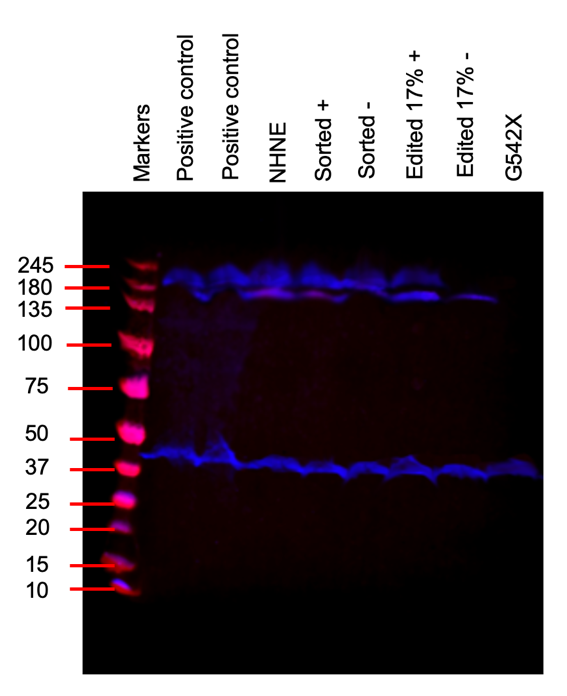


b.


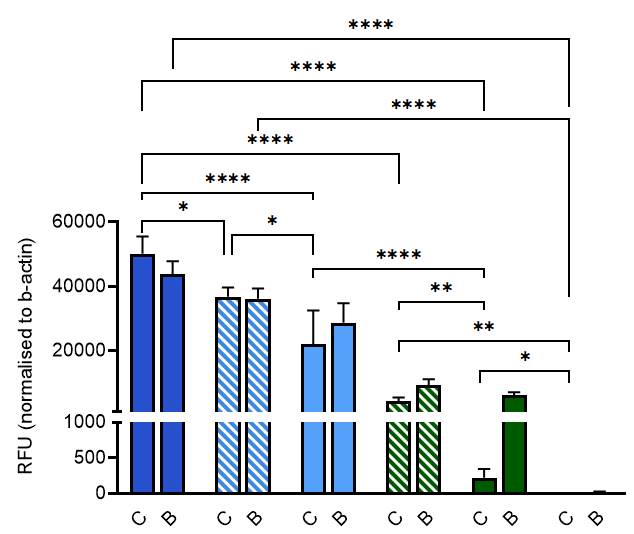

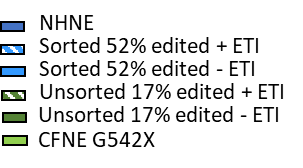


Figure S6. a. Representative full uncropped image of western blot showing abundance of CFTR bands B and C. Positive control was protein (20μg) extracted from Fisher Rat Thyroid cells, expressing CFTR, grown at liquid-liquid-interface (kind gift E. Sorcher, Emory University, Atlanta, USA. All other samples as outlined in manuscript Figure 3b. b. Quantification of CFTR bands B and C obtained from representative images as shown in a. (manuscript Figure 3b). Quantification is shown as relative fluorescent units (RFU) obtained for CFTR band C/β-actin and band B/β-actin using OdysseyCLx for samples extracted from NHNE, unedited (CFNE G542X), edited (Unsorted 17%) and edited and FACs sorted (Sorted 52%) cells grown at ALI and treated with vehicle (-ETI) or ETI (+ETI). Data are shown as mean ± SD, n = 3. Significantly different as shown, * = p <0.05, ** = p < 0.01, *** = p <0.001, **** = p<0.0001, n=6.

Supplementary Figure S7 Short circuit current traces.

a.


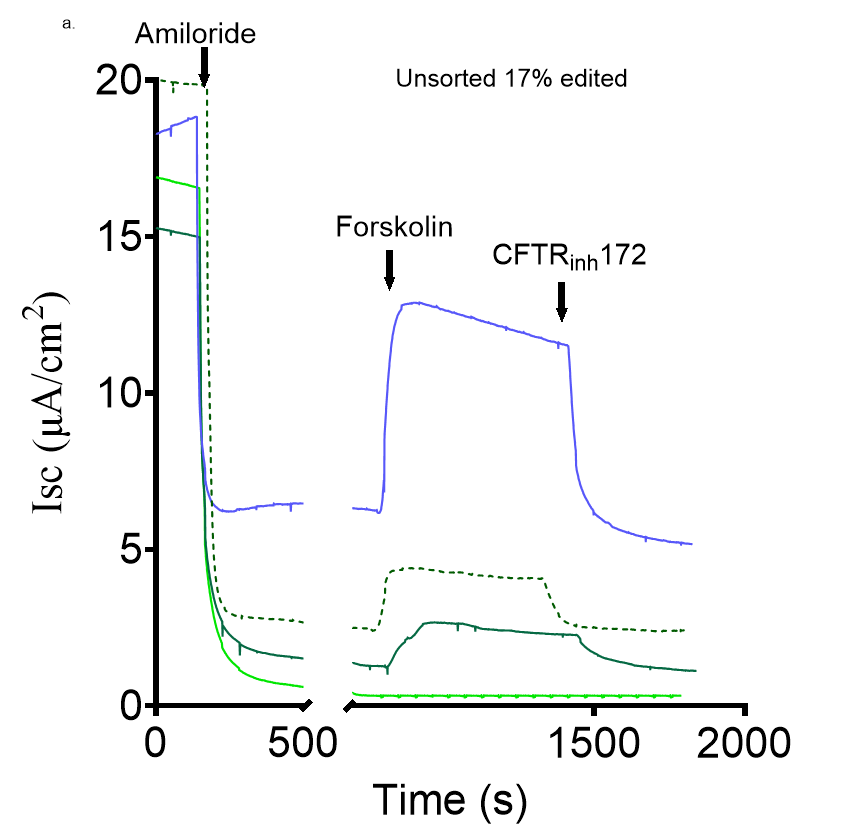


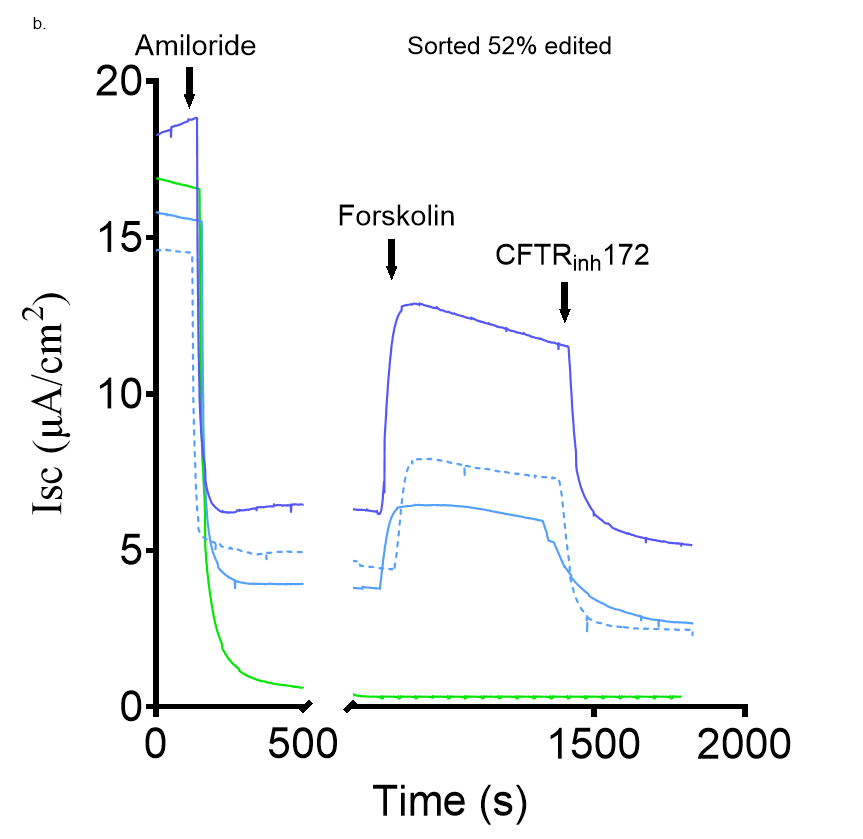

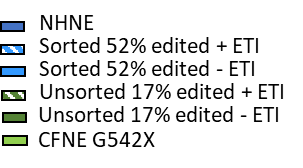


Figure S7. Representative short circuit current (I_sc_) traces from NHNE, CFBE G542X, unsorted 17% and sorted 52% cells after addition of amiloride (10 μM, apical), forskolin (10 μM, bilateral), CFTR inhibitor 172 (10 μM, apical) as indicated. a. I_sc_ traces from unsorted 17% before and after treatment with vehicle (-ETI) or ETI (+ETI). b. sorted 52% cells before and after treatment with vehicle (-ETI) or ETI (+ETI) Untreated NHNE and CFBE G542X are the same on both graphs for reference.

Supplementary Figure S8 Ciliated and secretory cell abundance.

a.


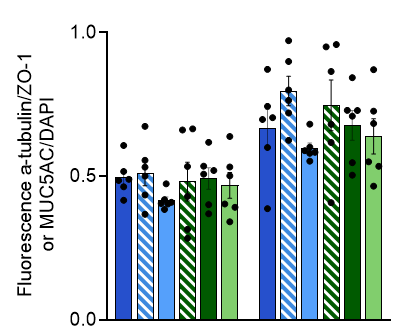

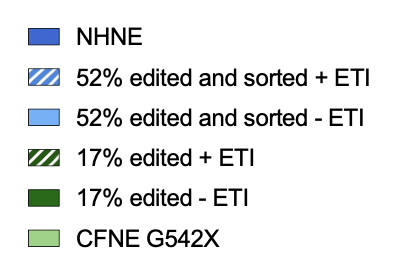


b.


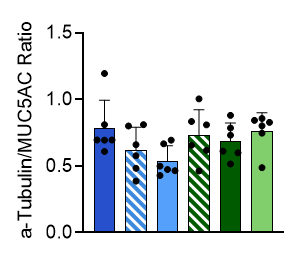


Figure S8. Quantification of α-tubulin labelled cells (ciliated) or MUC5AC labelled cell (secretory) obtained from images as represented in Figure 6. a. Quantification is shown as relative fluorescent units (RFU) obtained from ImageJ for α-tubulin fluorescence/ZO-1 or MUC5AC/DAPI for n=6 images. Data are shown as mean ± SD.
